# Water-Pipe Smoking and Metabolic Syndrome: A Population-Based Study

**DOI:** 10.1371/journal.pone.0039734

**Published:** 2012-07-27

**Authors:** Kashif Shafique, Saira Saeed Mirza, Muhammad Kashif Mughal, Zain Islam Arain, Naveed Ahmed Khan, Muhammad Farooq Tareen, Ishtiaque Ahmad

**Affiliations:** 1 Institute of Basic Medical Sciences, Dow University of Health Sciences, Karachi, Pakistan; 2 Department of Medicine, Isra Medical University Hospital, Hyderabad, Pakistan; 3 Department of Surgery, Civil Hospital Karachi, Karachi, Pakistan; 4 Department of Health, Government of Pakistan; 5 Afra General Hospital, Faisalabad, Pakistan; University of Oxford, United Kingdom

## Abstract

Water-pipe (WP) smoking has significantly increased in the last decade worldwide. Compelling evidence suggests that the toxicants in WP smoke are similar to that of cigarette smoke. The WP smoking in a single session could have acute harmful health effects even worse than cigarette smoking. However, there is no evidence as such on long term WP smoking and its impact on chronic health conditions particularly cardiovascular and metabolic conditions. Therefore, we conducted this study to investigate the relationship between WP smoking and metabolic syndrome (MetS). This was a cross-sectional study carried out in Punjab province of Pakistan using the baseline data of a population-based study – Urban Rural Chronic Diseases Study (URCDS). Information was collected by trained nurses regarding the socio-demographic profile, lifestyle factors including WP smoking, current and past illnesses. A blood sample was obtained for measurement of complete blood count, lipid profile and fasting glucose level. MetS was ascertained by using the International Diabetic Federation’s criteria. We carried out multiple logistic regressions to investigate the association between WP smoking and MetS. Final sample included 2,032 individuals – of those 325 (16.0%) were current WP smokers. Age adjusted-prevalence of MetS was significantly higher among current WP smokers (33.1%) compared with non-smokers (14.8%). Water-pipe smokers were three times more likely to have MetS (OR 3.21, 95% CI 2.38–4.33) compared with non-smokers after adjustment for age, sex and social class. WP smokers were significantly more likely to have hypertriglyceridemia (OR 1.63, 95% CI 1.25–2.10), hyperglycaemia (OR 1.82, 95% CI 1.37–2.41), Hypertension (OR 1.95, 95% CI 1.51–2.51) and abdominal obesity (OR 1.93, 95% CI 1.52–2.45). However, there were no significant differences in HDL level between WP smokers and non-smokers. This study suggests that WP smoking has a significant positive (harmful) relationship with MetS and its components.

## Introduction

Water-pipe (WP; shisha, narghile, hookah and arghile) is a four century old tobacco use device [1], [2]. In a typical WP configuration, perforated aluminium foil separates burning charcoal from flavoured tobacco that is attached to a water bowl. A hose attached to the water bowl is used to draw air through the mouthpiece of the hose which causes the tobacco and charcoal smoke to bubble through the water whereupon they are inhaled by the smoker [1], [2]. As the smoke passes through water, it is believed that smoke is “filtered” by the water and its harmful effect is reduced by the so-called “filtering” process [3], [4]. Traditionally used WP was fairly identical to the modern and fashionable WP except the fact that tobacco and charcoal were used in very raw form without any added flavouring agents, however a piece of raw brown sugar piece was also installed in WP device just beneath the charcoal bowl to have a sweet flavouring effect in the smoke.

WP smoking is becoming increasingly popular worldwide particularly among younger age groups [2], [5]. The highest prevalence of current WP smoking was among school students across countries: the United States, especially among Arab Americans (12%–15%) the Arabic Gulf region (9%–16%), Estonia (21%), and Lebanon (25%) [2], [5]. Furthermore, the prevalence of WP smoking was even higher among the university students, 25.5% in Syrian young men [6] and 24.5% in the United States [7]. In Pakistan, approximately half of the University students reported the ever-use of WP and 33% were current WP smokers [8].

Substantial body of evidence now suggests that WP contains many of the similar toxicants as cigarette smoke particularly the carbon monoxide (CO). Indeed, the CO content in WP smokers has been reported three to ten times higher compared with cigarette smokers following the smoking sessions [1], [9], [10]. Most of the CO in the mainstream of WP smokers is likely to originate from the burnt charcoal which is used in these smoking devices. Furthermore, the nicotine level of WP smokers has also been reported to be significantly higher than the cigarette smokers [11].

Given the harmful nature of WP smoke, impact on human health may be similar or even worse than cigarette smoking. Limited evidence shows that WP smoking has harmful acute effects on cardio-respiratory system [12], [13]. However, there is no evidence as such on the role of long-term WP smoking in chronic diseases particularly cardiovascular diseases and metabolic syndrome. Smoking has been consistently linked with cardiovascular disease, recent evidence suggests that metabolic syndrome is the potential link between cigarette smoking and cardiovascular diseases [14]. Metabolic syndrome is a cluster of risk factors which have shown a strong relationship with the risk of cardiovascular diseases. Individuals with the syndrome are twice as likely to die from and three times more likely to have a heart attack or stroke compared with people without the syndrome [15]. If the toxicants in WP smoke are fairly similar to those of smoking, then we might expect a relationship between WP smoking and metabolic syndrome. Therefore, the present study was conducted to examine the relationship between WP smoking and metabolic syndrome in apparently healthy population.

## Methods

### Cohort selection and study participants

We carried out a cross-sectional study using the baseline data of Urban Rural Chronic Diseases Study (URCDS). The details of this cohort have been described elsewhere [16]. In brief, this population-based cohort was established to understand the aetiology of emerging chronic diseases in Pakistan. This cohort was setup in the northeast of Punjab province in Faisalabad city (an urban centre) and included one peripheral area (a rural centre) which is approximately 35 kilometres away from the main city. This city has a population of approximately six million, making it the third largest city in the country. The study protocol was assessed and approved by Ethical Review Committee, Afra General Hospital, Faisalabad, Pakistan and the study was conducted in accordance with the Declaration of Helsinki guidelines. Study protocol was also approved by the three involved institutions – Afra General Hospital, Haider Medicare Hospital and New Lahore Hospital. All the individuals provided written informed consent before their participation.

Potential participants for this study were invited through a variety of methods; by sending personalised invitations, invitations through public gatherings, conveying the message to the community by key informants, local political leaders and message by the religious leaders through the mosque.

Only, apparently healthy individuals between the aged 30 to 75 years were included in this study. The participants visited the specially set-up clinic in three different centres during the period of 1^st^ January 2006 to 31^st^ June 2009. Participants included men and women, working or non-working and also those who had retired from their jobs. Individuals who were accompanying a patient for routine health check-ups were also invited and if agreed, were included in this study.

Study protocol comprised of an interview by a trained nurse at site followed by a screening examination by a physician at a specially set-up clinic. Questions included demographic details, occupation, lifestyle habits including smoking, and health. Social class was assigned to each participant on the basis of their occupation. Upper-class was professionals (those with executive jobs in the government or private sectors), middle-class was non-manual workers (skilled individuals with office jobs) and lower-class was manual workers (unskilled workers or farmers). Non-working women were assigned the social class of their husbands or parents (if unmarried). As part of the screening examination, measurements were made for height, weight, waist circumference and blood pressure. A blood sample was obtained at baseline screening for the measurement of lipid profile and glucose level. A subset of participants was also investigated for C-reactive protein and Microalbuminuria.

### Metabolic syndrome definition

We used the new International Diabetes Federation (IDF) definition for the ascertainment of MetS. According to the new definition for a person to be defined as having the MetS must have central obesity plus any two of the four factors which include raised triglyceride (TG) (≥1.7mmol/L or specific treatment for this lipid abnormality), reduced High Density Lipoprotein (HDL) cholesterol (<1.03 mmol/L in males and <1.29 mmol/L in females or specific treatment for this lipid abnormality), raised blood pressure (systolic blood pressure ≥130 or diastolic blood pressure ≥85 or treatment of previously diagnosed hypertension) and raised fasting plasma glucose (≥5.6mmol/L or previously diagnosed type 2 diabetes) [15]. To determine the abdominal obesity we used South Asian specific cut off in which a waist circumference for male ≥ 90cm and female ≥80 was considered as obese [17].

### Sample size estimation

We estimated the sample size to measure 10% difference of MetS prevalence (assuming 30% prevalence of MetS among WP smokers) between groups at 0.05 significance level using two sided comparison and power of 80%. Sample size was computed for both the *X^2^* using the Yates’ continuity correction and Fisher Exact Test. A sample of participants 626 was the minimum number required to be accrued to perform this survey with an allocation ratio of 1.

### Data analysis

We used Stata software version 11 (StataCorp, College Station, TX, USA) to analyze the collected information. Participants were divided into two groups according to their WP smoking status (“water-pipe smokers” and “non-smokers”). Current water-pipe smokers were those who regularly used water-pipe once in a week in last 12 months. Non-smoker category comprised of those who never smoked. Ex-smokers were those who reported giving up smoking at least a year before baseline screening, otherwise they were defined as current smokers. Number of ex smokers was small (n = 13) so they were combined with never smokers to make a category. Age was categorised into 10 year age bands (age years 30–39,40–49,50–59 and 60–75). We created variables to estimate the age-adjusted prevalence of raised blood pressure, hypertriglyceridemia, low HDL level and raised glucose level using the IDF specific criteria, which include the raised level of these measures or a previously diagnosed and treated condition. Therefore, wherever hypertension, hypertriglyceridemia, low HDL or hyperglycaemia is mentioned in this manuscript that indicates a combination of increased level of the specified measure or a previously known condition. We used independent sample t test to compare continuous variables and chi-square test for the categorical variables. We used multiple logistic regressions to evaluate the association between WP smoking and components of MetS as well as overall existence of MetS. We ran separate multivariate logistic regression models for males and females to investigate the relationship between WP smoking and MetS after adjusting for age and social class.

Information was collected from 2700 individuals, however blood pressure data were missing for 75 participants, self-reported diabetes mellitus and blood glucose for 47, height and weight for 24, social class and triglycerides level for 79 individuals, therefore these participants were excluded from the analysis. Furthermore, those who were cigarette smokers or combine users of cigarette and WP (n = 443) were also excluded from the present analysis.

## Results

Data from 2,032 individuals were available for final analysis, 1,039 (51.1%) were males and 993 (48.9%) were females. Of these, 325 (16.0%) individuals were current WP smokers and 1,707 (84.0%) were non smokers. There were no statistically significant differences of age at screening, sex and mean HDL levels between WP smokers and non-smokers. However, significant differences of social class, waist circumference, serum TG levels, blood pressure and fasting glucose levels were observed between WP smokers and non-smokers. The mean duration of water-pipe smoking in this sample was 15.57±11.6 years, there was no significant difference in duration among males and females (p-vaule 0.17). WP smokers were more likely to be in the professional group (p-value <0.01), obese (p-value <0.01), higher waist circumference (p-value <0.01), an elevated TG level (p-value <0.01), raised systolic and diastolic blood pressure (p-value <0.01) and raised fasting glucose level (p-value <0.01). Baseline characteristics of study population are described in table 1.

**Table 1:** Baseline characteristics of water-pipe smokers and non-smokers.

	Water-pipe smokers	Non smokers	P-value
	(n = 325)	(n = 1,707)	
	n (%) *	n (%) *	
**Age at screening (years)**			
30–39	64 (19.7)	419 (24.6)	0.05
40–49	77 (23.7)	464 (27.2)	
50–59	80 (24.6)	359 (21.0)	
60–75	104 (32.0)	465 (27.2)	
**Sex**			
male	175 (53.8)	864 (50.6)	0.29
female	150 (46.2)	843 (49.4)	
**Social class**			
Professional	71 (21.9)	252 (14.8)	<0.01
Non-manual workers	137 (42.1)	659 (38.6)	
Manual workers	117 (36.0)	796 (46.6)	
**Waist circumference (cm), mean(s.d.)**	84.7 (12.6)	80.6 (11.8)	<0.01
**HDL level, mean (s.d.)**	1.3 (0.41)	1.3 (0.40)	0.24
**Serum triglyceride level, mean (s.d.)**	1.6 (1.0)	1.4 (0.90)	<0.01
**Blood pressure mmHg, mean (s.d.)**			
Systolic	129.9 (21.9)	124.4 (19.7)	<0.01
Diastolic	74.3 (9.9)	71.9 (9.6)	<0.01
**Fasting glucose level mmols/l, mean (s.d.)**	5.2 (1.7)	4.9 (1.18)	<0.01

Table presents numbers and percentage until stated otherwise.

Using the International Diabetes Federation cut offs for the components of MetS, the age-adjusted proportions of participants who had a raised level of TG (or specific treatment for lipid disorder), raised systolic blood pressure (or previously diagnosed hypertension and receiving treatment) and central obesity also differed significantly between WP smokers and non-smokers. Overall age-adjusted prevalence of MetS was significantly higher (p-value <0.01) among WP smokers (33.1%) compared with non-smokers (14.8%).

On logistic regression analysis, WP smokers were significantly more likely to have hypertriglyceridemia (OR 1.63, 95% CI 1.25–2.10), hyperglycaemia (OR 1.82, 95% CI 1.37–2.41), Hypertension (OR 1.95, 95% CI 1.51–2.51) and abdominal obesity (OR 1.93, 95% CI 1.52–2.45) (table 2). However, there were no significant differences in HDL level between WP smokers and non-smokers. Furthermore, sex stratified analysis showed that male WP smokers were significantly more likely to have a low HDL level (OR 1.75, 95% CI 1.11–2.78), hypertriglyceridemia (OR 1.60, 95% CI 1.06–2.42) and hyperglycaemia (OR 1.88, 95% CI 1.22–2.89) (table 2). However, there were no significant differences in hypertension and central obesity between male WP smokers and non-smokers (table 2). Female WP smokers were more likely to have hypertriglyceridemia, hypertension and central obesity, while there were no significant differences in HDL level and glucose levels (table 2).

**Table 2:** Relationship between water-pipe smoking and components of metabolic syndrome.

				Low HDL Level*			Hypertriglyceridemia*		Hyperglycaemia*		Hypertension*		Central obesity		
*Characteristic*	Total participants, n(%)			Odds Ratio (95%CI)			Odds Ratio (95%CI)		Odds Ratio (95%CI)		Odds Ratio (95%CI)		Odds Ratio (95%CI)		
**Males**															
Non smokers	864	(83.1)		reference			reference		reference		reference		reference		
Smokers	175	(16.9)		1.75		(1.11, 2.78)	1.60	(1.06, 2.42)	1.88	(1.22, 2.89)	1.31	(0.86,2.01)	1.21	(0.81, 1.81)	
**Females**															
Non smokers	843	(84.9)		reference			reference		reference		reference		reference		
Smokers	150	(15.1)		1.13	(0.77, 1.67)		1.53	(1.06, 2.19)	1.21	(0.77, 1.90)	2.04	(1.35, 3.08)	2.31	(1.54, 3.46)	

All estimates were adjusted for age at screening, social class and area of residence. * indicate increased level of specific measure or a previously diagnosed condition.

On further analysis, male WP smokers were three times more likely to have MetS (OR 3.52, 95% CI 2.32–5.37) compared with non-smokers. Similarly, female WP smokers were three times more likely to have MetS (OR 3.41, 95% CI 2.37–4.89) (table 3). These associations changed a little after adjustment for age and social class and the overall association between WP smoking and MetS remained consistent, both in males and females (table 3). Age and social class also appeared as significant determinants of MetS in both univariate and multivariate analyses. The odds of having MetS increased with increase in age with the highest risk among oldest age group (age 60–75 years). Furthermore, non-manual and manual worker groups showed significantly lower odds of having MetS compared with the professional group (table 3).

**Table 3:** Relationship between water-pipe smoking and metabolic syndrome.

	Metabolic syndrome				Metabolic syndrome			
	Males				Females			
	Univariate analysis	P-value	Multivariate analysis	P-value	Univariate analysis	P-value	Multivariate analysis	P-value
	OR (95% CI)		OR (95% CI)		OR (95% CI)		OR (95% CI)	
**Water-pipe smoking status**								
Non smokers	reference		reference		reference		reference	
Current smokers	3.52 (2.32–5.37)	<0.01	3.16 (1.97–5.07)	<0.01	3.41 (2.37–4.89)	<0.01	3.28 (2.22–4.84)	<0.01
**Age at screening (years)**								
30–39	reference		reference		reference		reference	
40–49	5.57 (1.61–19.33)	<0.01	5.45 (1.55–19.18)	<0.01	2.60 (1.45–4.66)	<0.01	2.10 (1.14–3.87)	0.02
50–59	16.01 (4.83–53.00)	<0.01	13.98 (4.16–47.04)	<0.01	5.10 (2.88–9.05)	<0.01	4.45 (2.44–8.07)	<0.01
60–75	33.38 (10.33–107.82)	<0.01	27.03 (8.26–88.51)	<0.01	5.47 (3.16–9.47)	<0.01	4.91 (2.78–8.67)	<0.01
**Social class**								
Professional	reference		reference		reference		reference	
Non-manual workers	0.39 (0.25–0.62)	<0.01	0.51 (0.32–0.83)	<0.01	0.35 (0.24–0.52)	<0.01	0.35 (0.23–0.53)	<0.01
Manual workers	0.10 (0.06–0.19)	<0.01	0.16 (0.08–0.29)	<0.01	0.16 (0.10–0.24)	<0.01	0.16 (0.11–0.26)	<0.01

Multivariate models include all co-variates presented in the table.

## Discussion

This population-based study demonstrated a significant positive (harmful) relationship between WP smoking and MetS. The observed relationship between WP smoking and MetS was independent of age, sex, social class and area of residence. The components of MetS also showed a significant relationship with WP smoking. In males, MetS among WP smokers was mainly driven by the impaired lipid profile and hyperglycaemia, while in female WP smokers, central obesity and hypertension were the major components contributing towards the development of MetS.

The adverse health effects of WP smoking largely remain unknown and the existing evidence is of very low to low quality. There is some evidence demonstrating that WP smoking significantly increases the risk of lung cancer and respiratory illness [18]; however, we are unaware of any published literature on chronic effects of WP smoking on the cardiovascular system. Two earlier studies investigated the acute effects of WP smoking on cardiovascular and respiratory systems. Study conducted on relatively younger age group showed that thirty minutes following a single WP smoking session, there were significant increases in the blood pressure, respiratory rate and heart rate of healthy individuals [13]. Another study from Israel also reported similar findings and interestingly, the harmful effects on cardio-respiratory system were even greater among female users compared with males [12].

Our findings are biologically plausible in many ways. Foremost, given the existence of similar toxicants in WP as cigarettes, components of MetS may have been mediated by the WP smoking. The main pathways which may be involved are the insulin resistance caused by toxic effects of CO either through the alteration in the body fat or by exerting a direct toxic effect on pancreatic tissue [14]. Moreover, Nicotine can also contribute to increase the abdominal obesity by activating the hypothalamic-pituitary-adrenal (HPA) axis [19]. Cortisol level was found significantly higher among cigarette smokers compared with non-smokers [19]. Thus, the hyperactivity of HPA axis and higher cortisol secretion observed among smokers due to nicotine may be responsible for the increase in abdominal obesity among smokers [19]. The impairment of lipid profile of WP smokers may also have been influenced by nicotine. Nicotine has been proposed to stimulate release of catecholamines by increasing sympathetic nerve activity. This leads to lipolysis and increased plasma concentration of TG, in which enzymes were considered involved in alterations of TG and HDL-C metabolism [20]. Some evidence also showed that elevated hepatic lipase activity among smokers can lead to a reduction of HDL-C and increased TG [21]. In addition, increased hepatic re-esterification of free fatty acids through enhanced lipolysis by smoking tended to increase hepatic very low-density lipoprotein (VLDL)-TG production and resulted in increased TG [21].

Our results may have been influenced by some biases. There is a possibility of a bias caused by not including the individuals who were smokers of both WP and cigarette in the present study. Differential dietary intake and physical activity may have confounded the apparent relationship between WP smoking and MetS. Although we made adjustment for social class which reflects some of these lifestyle factors, however, some of the residual confounding may still have influenced our results. Furthermore, BMI was also included in the multivariate model as another proxy measure of dietary intake and physical activity but accounting for BMI also did not alter the relationship between WP smoking and MetS (data not shown). Given that WP is smoked in gatherings with few people sitting around, exposure to second-hand smoke may have some influence on our results. Some of the non-smokers may have been exposed to second-hand smoke through contaminated air and resulted in impairment in their metabolic profile as well. If this was true, it is unlikely to affect the observed relationship of WP smoking and MetS, however, the effects of WP observed in this study may have been attenuated and true effect may even be stronger than the observed effect.

The major strength of this study is its population-based design and a fairly large sample to examine relationships between WP smoking and MetS. This study has several limitations which need to be mentioned. First, the study sample was only obtained from one province of Pakistan; the findings of the present study may not be generalisable to other regions of this country. This is particularly important because the distribution of cardio-metabolic risk factors and conditions significantly varies between different ethnic groups residing in this country. Study sample was obtained using variety of different approaches to increase the participation rate, this could have introduced some selection bias however an earlier analysis suggests that prevalence of cardiovascular risk factors (i.e. obesity, hypertension and diabetes mellitus) is fairly consistent with other reports from this region so selection bias may not be a serious concern in relation to present findings. Second, although our findings suggest an association between WP smoking and MetS – the cross sectional design used in this study cannot determine the causal role of WP smoking in the development of MetS. Finally, participants of this study used to smoke WP prepared in the traditional way i.e. using raw tobacco and charcoal which may not be absolutely identical in terms of smoke toxicants which are produced by the modern WP available in public cafes. However, earlier evidence has shown that flavouring of tobacco and charcoal does not significantly reduce the harmful effect of WPs.

### Public health implications

WP smoking has remarkably increased worldwide particularly among younger individuals. Given the harmful effect observed in this study and earlier reported elsewhere, WP smoking will pose significant burden on morbidity and mortality due to chronic diseases. Confirmation of causality between WP smoking and metabolic conditions is necessary so that wider public health measures against its widespread use can be initiated. Further research with longitudinal design including the younger age group and different types of tobacco’s use in the market may provide valuable insights on this issue and guide the public policy development. Health education and awareness are perhaps the most crucial interventions required to be delivered so that the false perceptions about its harmlessness can be changed and the adverse effects of the WP smoking could be appreciated by the community. These interventions need to be focused on all age groups, particularly in younger age groups to avoid future morbidity and mortality associated with WP smoking.

In conclusion, Water-pipe smoking has significant independent relationship with metabolic syndrome among middle and old age individuals. Further evidence is required to understand the mechanism and establish the temporal relationship between WP and MetS.
